# Downregulation of ATP6V1A Involved in Alzheimer's Disease via Synaptic Vesicle Cycle, Phagosome, and Oxidative Phosphorylation

**DOI:** 10.1155/2021/5555634

**Published:** 2021-04-19

**Authors:** Zhike Zhou, Jun Bai, Shanshan Zhong, Rongwei Zhang, Kexin Kang, Xiaoqian Zhang, Ying Xu, Chuansheng Zhao, Mei Zhao

**Affiliations:** ^1^Department of Geriatrics, The First Affiliated Hospital, China Medical University, Shenyang, 110001 Liaoning, China; ^2^Cancer Systems Biology Center, The China-Japan Union Hospital, Jilin University, Changchun, 130033 Jilin, China; ^3^Department of Neurology, The First Affiliated Hospital, China Medical University, Shenyang, 110001 Liaoning, China; ^4^Computational Systems Biology Lab, Department of Biochemistry and Molecular Biology and Institute of Bioinformatics, The University of Georgia, USA; ^5^Department of Cardiology, The Shengjing Affiliated Hospital, China Medical University, Shenyang, 110004 Liaoning, China

## Abstract

**Objective:**

The objective of this study was to investigate the potential molecular mechanisms of ATPase H^+^ transporting V1 subunit A (ATP6V1A) underlying Alzheimer's disease (AD).

**Methods:**

Microarray expression data of human temporal cortex samples from the GSE118553 dataset were profiled to screen for differentially expressed genes (DEGs) between AD/control and ATP6V1A-low/high groups. Correlations of coexpression modules with AD and ATP6V1A were assessed by weight gene correlation network analysis (WGCNA). DEGs strongly interacting with ATP6V1A were extracted to construct global regulatory network. Further cross-talking pathways of ATP6V1A were identified by functional enrichment analysis. Diagnostic performance of ATP6V1A in AD prediction was evaluated using area under the curve (AUC) analysis.

**Results:**

The mean expression of ATP6V1A was significantly downregulated in AD compared with nondementia controls. A total of 1,364 DEGs were overlapped from AD/control and ATP6V1A-low/high groups. Based on these DEGs, four coexpression modules were predicted by WGCNA. The blue, brown, and turquoise modules were significantly correlated with AD and low ATP6V1A, whose DEGs were enriched in phagosome, oxidative phosphorylation, synaptic vesicle cycle, focal adhesion, and gamma-aminobutyric acidergic (GABAergic) synapse. Global regulatory network was constructed to identify the cross-talking pathways of ATP6V1A, such as synaptic vesicle cycle, phagosome, and oxidative phosphorylation. According to the AUC value of 74.2%, low ATP6V1A expression accurately predicted the occurrence of AD.

**Conclusions:**

Our findings highlighted the pleiotropic roles of low ATP6V1A in AD pathogenesis, possibly mediated by synaptic vesicle cycle, phagosome, and oxidative phosphorylation.

## 1. Introduction

Alzheimer's disease (AD), referring to a progressive neurodegenerative disease, is pathologically characterized by extracellular senile plaques composed of amyloid beta (A*β*), neurofibrillary tangles composed of hyperphosphorylated tau, and neuron loss [1, 2]. In brain parenchyma of AD, A*β* peptide is derived from continuous cleavage of amyloid precursor proteins (APP) by *β*- and *γ*-secretases, with its deposition depending on the balance between production and removal [3, 4]. As the major processing compartment for A*β*, lysosomes rely on an acidic environment of pH less than 5.0 to activate proteases for A*β* degradation [4, 5]. This gradient of acidification is potentially mediated by vacuolar H^+^-ATPase (V-ATPase), a multisubunit enzyme consisting of V0 and V1 sectors that pumps protons into the lysosomal lumen by ATP consumption [6]. Dysfunction of V-ATPase-dependent acidification disrupts the trafficking of substrates between endolysosomal compartments, which may facilitate molecular steps for neuronal degeneration, such as AD [7].

ATPase H^+^ transporting V1 subunit A (ATP6V1A) encoding a peripheral subunit in V1 sector of V-ATPase constitutes ATP-binding interface and catalyzes ATP hydrolysis [8]. The resultant energy is then coupled with a ring of proteolipid subunits in V0 sector, giving rise to proton translocation across the membrane [9]. For instance, ATP6V1A assembles with V0 subunits to form the glucose-dependent complex of V1/Vo sectors that drives proton transport; simultaneously, this process can be interrupted by reversible dissociation of the complex into component V0 and V1 [10]. Several lines of evidence have demonstrated that misrouting V0 subunit-induced proton translocation contributed to defective lysosomal acidification, a devastating manifestation in virtually all lysosomal storage and neurodegenerative diseases [11–13]. However, the pathophysiological mechanism of AD due to dysfunction of V1 subunits (e.g., ATP6V1A) remains elusive and is difficult to be verified by traditional biological methods. Accordingly, we sought to perform a comprehensive bioinformatics analysis of ATP6V1A based on gene expression data and functional annotations, aiming to elucidate the molecular functions of ATP6V1A underlying the pathogenesis of AD.

## 2. Materials and Methods

### 2.1. Data Resources

The RNA microarray data of human postmortem temporal cortex samples, including 45 AD patients and 24 nondementia controls, were downloaded from the GSE118553 dataset of Gene Expression Omnibus (GEO, https://www.ncbi.nlm.nih.gov/geo/) [14]. This dataset was analyzed using Illumina HumanHT-12 V4.0 expression beadchip on the platform of GPL10558. Taking the average expression of ATP6V1A as the cut-off line, enrolled samples were dichotomized into two groups: the ATP6V1A-low group and the ATP6V1A-high group. Similarly, samples were divided into age-low/high groups according to the cut-off line of the mean age. The odds ratio (OR) was calculated by logistics regression analysis to detect the potential predictors of AD (Table 1). Low-expressed probes were removed to retain the highest one if a gene corresponded to multiple probes. Gene expression profiles were preprocessed by normalization adopting the *normalizeBetweenArrays* function in the *limma* package of R software version 3.6.2 [15].

### 2.2. Gene Set Enrichment Analysis (GSEA)

GSEA analysis was conducted to screen out the biological processes (BP) of gene ontology terms that were significantly enriched in phenotypes of AD and low ATP6V1A expression [16, 17]. The number of permutations was set to 1000 and normalized *P* < 0.05 was considered for significant enrichment. The results of GSEA were visualized by using *ClusterProfler*, *enrichplot*, *ggplot2*, and *GSEABase* packages.

### 2.3. Differential Expression Analysis

The *lmFit* and *eBayes* functions in *limma* packages were used to filtrate differentially expressed genes (DEGs) between AD/control and ATP6V1A-low/high groups. Analyses of two-dimensional hierarchical clustering and volcano plot were conducted employing the *limma* package in R. Threshold of statistical significance was defined as fold change (FC) ≥ 1.5 and a false discovery rate- (FDR-) adjusted *P* < 0.05 [15, 18, 19].

### 2.4. Coexpression Network Analysis

Data of DEGs overlapped from AD/control and ATP6V1A-low/high groups were processed by weight gene correlation network analysis (WGCNA). Outlier samples were eliminated in clustering dendrogram to ensure the reliable outcome of coexpression network using the *hclust* function. The soft thresholding power of 6 was selected by *pickSoftThreshold* function to make the network conform to the power-law distribution and close to the real state of biological network [20]. Clustering tree was dissected into branches to construct coexpression modules of at least 30 genes, which were visualized by different color labels [21, 22]. The *labeledHeatmap* function was used to display the correlation values within a heatmap plot of module-trait relationships. Functional enrichment analysis of Kyoto Encyclopedia of Genes and Genomes (KEGG) pathways were performed using *clusterProfiler* package.

### 2.5. Construction of Global Regulatory Network and Cross-Talking Pathways of ATP6V1A

The intramodular connectivity and genetic phenotype were measured by module membership (MM) and gene significance (GS), respectively. Scatter diagram of the relationship between MM and GS was plotted using *verboseScatterplot* function [23]. According to the empirical criteria of MM > 0.4 and GS > 0.5, DEGs strongly interacting with ATP6V1A expression were extracted, thus to construct global regulatory network of proteins based on the STRING database (Search Tool for the Retrieval of Interacting Genes, https://www.string-db.org/) [24]. The cross-talking pathways of ATP6V1A were identified by functional enrichment analysis of KEGG pathways. Visualization of global regulatory network and cross-talking pathways of ATP6V1A was accomplished adopting *cytoscape* software [25].

### 2.6. Signature Genes of a Pathway

The correlation between genes was quantitatively determined by Pearson correlation coefficient (PCC) analysis [26]. For each cross-talking pathway, the five genes with the highest PCC were designated as signature genes, whose expression was most strongly correlated with other genes of the pathway [27]. If the signature genes of a pathway were significantly correlated with ATP6V1A expression, ATP6V1A was recognized to modulate or mediate the pathway.

### 2.7. Analysis of Area under the Curve (AUC)

The *pROC* function was used to estimate diagnostic performance of ATP6V1A in distinguishing AD from nondementia. Under continuous threshold conditions, classification performance was displayed via receiver operating characteristic (ROC) curves, and the discrimination was quantified by measuring AUC. As a method widely applied in medical diagnostics, AUC analysis estimated the probability of a model that could accurately differentiate the occurrence of events among randomly selected individuals [28]. An AUC value of 100% was for complete prediction and 50% for random selection. All *P* values were bilateral, and statistical significance at *P* < 0.05 was selected.

### 2.8. Cell-Type Proportion Analysis

To assess the proportion of cell types in the bulk tissue RNA-seq data in our study, a cell-type deconvolution on each sample was conducted by the brain cell-type marker signatures provided by the *BRETIGEA* R package [29]. Each cell type (e.g., neurons, endothelial cells, oligodendrocytes, microglia, astrocytes, and oligodendrocyte precursor cells (OPCs)) using 1000 marker genes from the human brain cell marker gene set was computed to generate an estimate of all surrogate cell-type proportion values (SPVs). Adopting the default parameters and the SPVs calculated above, the bulk RNA-seq data was normalized by *BRETIGEA* function.

### 2.9. Cell-Type Specificity Plot

To generate the cell-type specificity plot, each squared expression was calculated as a vector and plotted from the center on a polar coordinate system based on the mean cell-type gene expression from Zhang et al. [30]. Thereafter, the vector sum of expression values for each gene were measured and multiplied by a scaling coefficient to form a final point as an estimate of the cell-type specificity for any gene under consideration.

## 3. Results

### 3.1. Baseline Characteristics of Samples and Identification of DEGs

**Figure 1:**
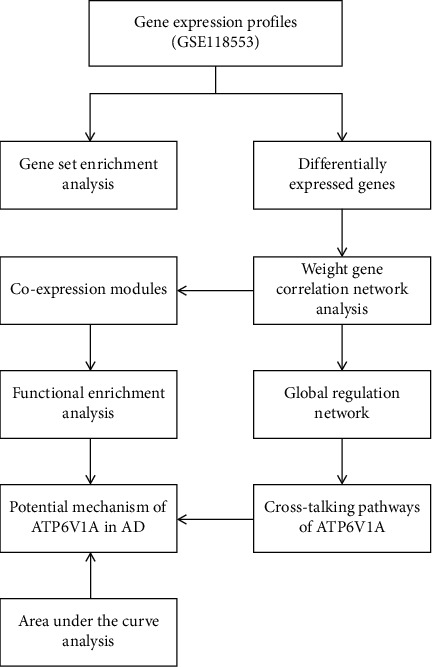
The flowchart of study design. AD: Alzheimer's disease.

**Figure 2:**
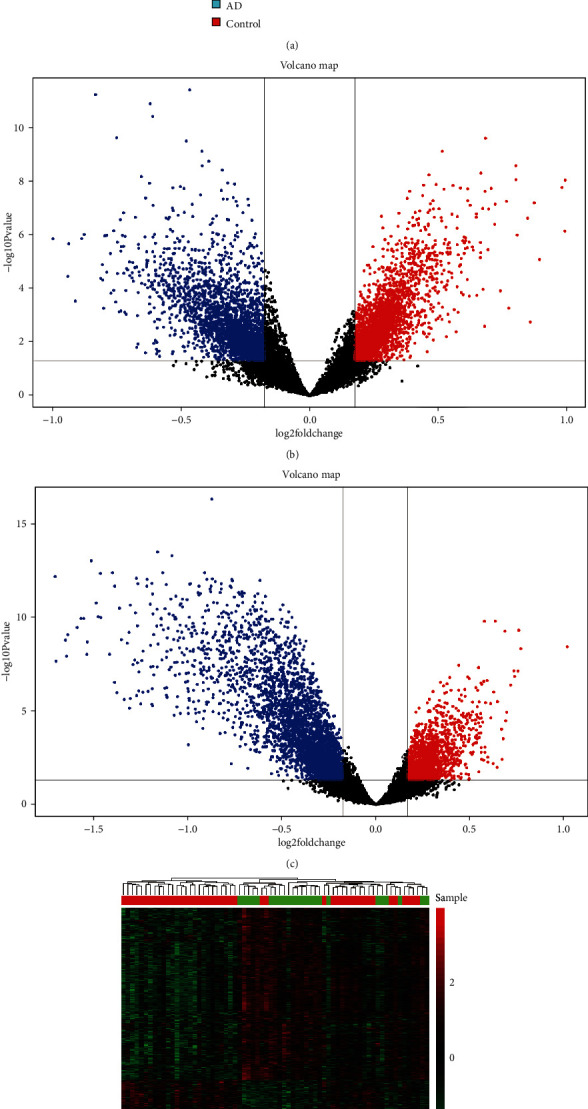
Differential expression gene analysis. ATP6V1A expression between AD and nondementia controls (a). Volcano plot of DEGs in the AD/control (b) and ATP6V1A-low/high groups (c): red represents upregulated and blue indicates downregulated. Heatmap of the DEGs in AD/control groups (d): green to red indicates the progression of gene expression from downregulated to upregulated. ^∗∗^*P* < 0.01; AD: Alzheimer's disease; DEGs: differentially expression genes.

The flowchart of study design was shown in Figure 1. The mean expression of ATP6V1A in the temporal cortex of AD (8.04 ± 0.41) was significantly lower than that of nondementia controls (8.42 ± 0.46; *P* = 0.001) (Figure 2(a)). This was consistent with western blot (Supplementary Figures 1A and 1B) and qRT-PCR (Supplementary Figure 1C) analyses of ATP6V1A expression between Mount Sinai Brain Bank (MSBB) Brodmann area 36 parahippocampal gyrus (BM36-PHG) samples of AD and normal controls [31]. Logistics regression analysis revealed that old age (OR = 0.246; *P* = 0.026) and low ATP6V1A expression (OR = 5.831; *P* = 0.003) were causally related to AD (Table 1). After removing unannotated and duplicate genes, 20,759 background genes were included for differential expression analysis. There were 3,416 DEGs (1,675 up- and 1,741 downregulated genes) filtrated in AD versus nondementia controls (Figure 2(b)). Whilst 5,303 DEGs (1,820 up- and 3,483 downregulated genes) were differentially expressed in ATP6V1A-low compared with high cohort (Figure 2(c)). Finally, 1,364 DEGs (577 up- and 787 downregulated genes) were overlapped from AD/control and ATP6V1A-low/high groups. Heatmap of the DEGs between AD and nondementia controls was plotted in Figure 2(d).

### 3.2. Coexpression Modules and Functional Enrichment Analysis

**Figure 3:**
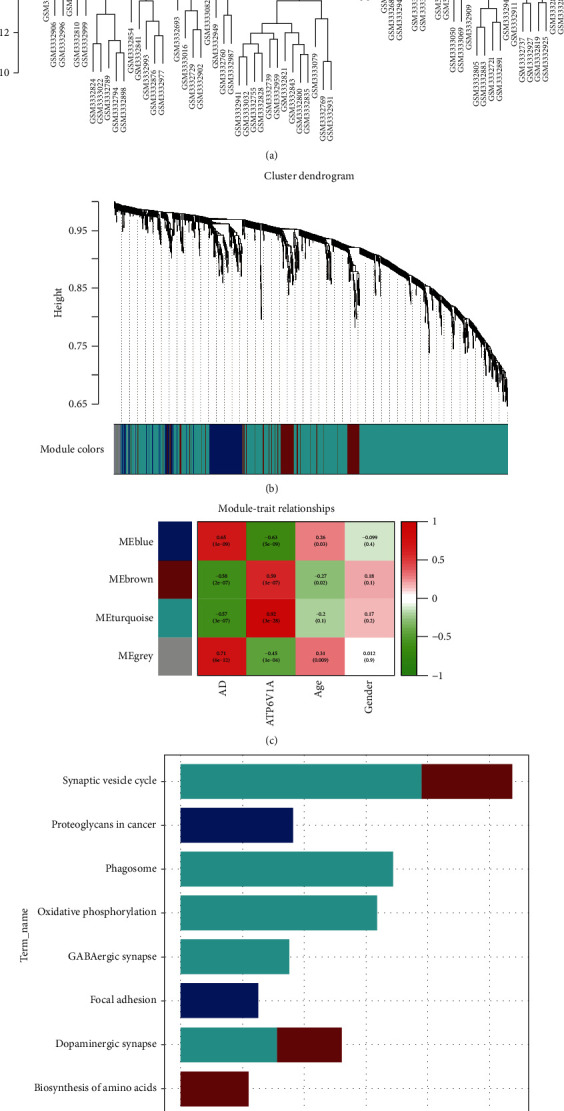
Weighted correlation network analysis. Clustering plot of samples (a). Cluster dendrogram of DEGs for distributing module colors (b): grey indicates nonclustering genes. Module-trait relationships of each module (c): green to red represents the correlation of modules from negative to positive with phenotypes. KEGG pathways of DEGs in each module (d). AD: Alzheimer's disease; KEGG: Kyoto Encyclopedia of Genes and Genomes.

All samples passed the cut-off line with a height of 25 and were hierarchically clustered by the average linkage (Figure 3(a)). Four coexpression modules (Figure 3(b)) were established by WGGNA, among which the grey module composed of noncoexpressed genes was regarded as the invalid module. Heatmap of module-trait relationships (Figure 3(c)) showed that blue module was positively correlated with AD (correlation coefficient = 0.65, *P* = 1*e* − 09) and negatively associated with ATP6V1A (correlation coefficient = −0.63, *P* = 5*e* − 09), whereas brown and turquoise modules had a negative correlation with AD (brown: correlation coefficient = −0.58, *P* = 2*e* − 07; turquoise: correlation coefficient = −0.57, *P* = 3*e* − 07) and positive association with ATP6V1A (brown: correlation coefficient = 0.59, *P* = 1*e* − 07; turquoise: correlation coefficient = 0.92, *P* = 3*e* − 28). Annotation of KEGG pathway (Figure 3(d)) was accomplished by functional enrichment analysis, showing that the DEGs in blue module were involved in proteoglycans in cancer and focal adhesion; the DEGs of brown module participated in biosynthesis of amino acids, dopaminergic synapse, and synaptic vesicle cycle; the DEGs in turquoise module were enriched in phagosome, dopaminergic synapse, oxidative phosphorylation, synaptic vesicle cycle, and gamma-aminobutyric acidergic (GABAergic) synapse.

### 3.3. Global Regulation Network and AUC Analysis of ATP6V1A

**Figure 4:**
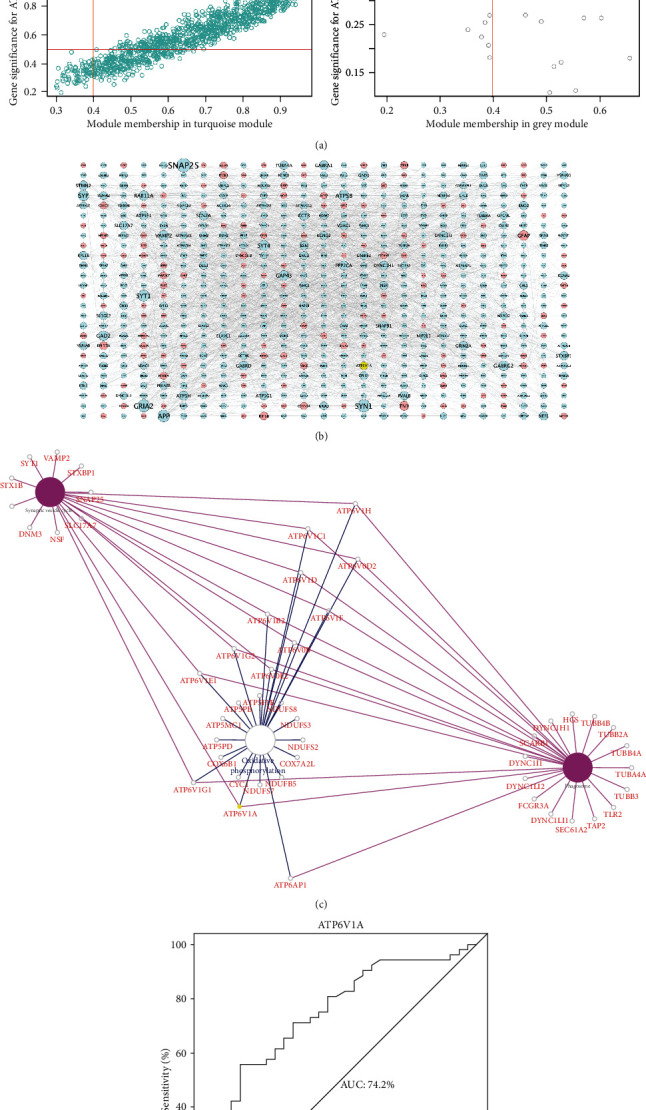
Module-pathway regulatory network and AUC analysis. Scatter diagram of module membership versus gene significance (a). Global regulatory network on DEGs interacting with ATP6V1A (b): red represents high expression; blue and yellow indicate low expression; the size of nodes reflects the degree of gene connectivity. Identification of cross-talking pathways of ATP6V1A (c): yellow indicates the low ATP6V1A expression. AUC analysis in AD prediction (d). AD: Alzheimer's disease; AUC: area under the curve.

As shown in Figure 4(a), scatter diagram between MM and GS (Figure 4(a)) showed a significant correlation between intramodular connectivity and genetic phenotypes in the blue, brown, and turquoise modules (blue: correlation coefficient = 0.71, *P* = 9.2*e* − 27; brown: correlation coefficient = 0.54, *P* = 6.9*e* − 12; turquoise: correlation coefficient = 0.94, *P* = 1*e* − 200), but not in the grey module (correlation coefficient = −0.31, *P* = 0.2). Thereafter, DEGs strongly interacting with ATP6V1A (MM > 0.4 and GS > 0.5) were extracted and displayed in the global regulation network (Figure 4(b)). Further cross-talking pathways of ATP6V1A, including phagosome, oxidative phosphorylation, and synaptic vesicle cycle, were identified (Figure 4(c)). The result of AUC analysis (AUC = 74.2%) exhibited a good diagnostic performance of low ATP6V1A expression in AD onset (Figure 4(d)).

### 3.4. Verification of ATP6V1A-Mediated Pathway and GESA in BP

**Figure 5:**
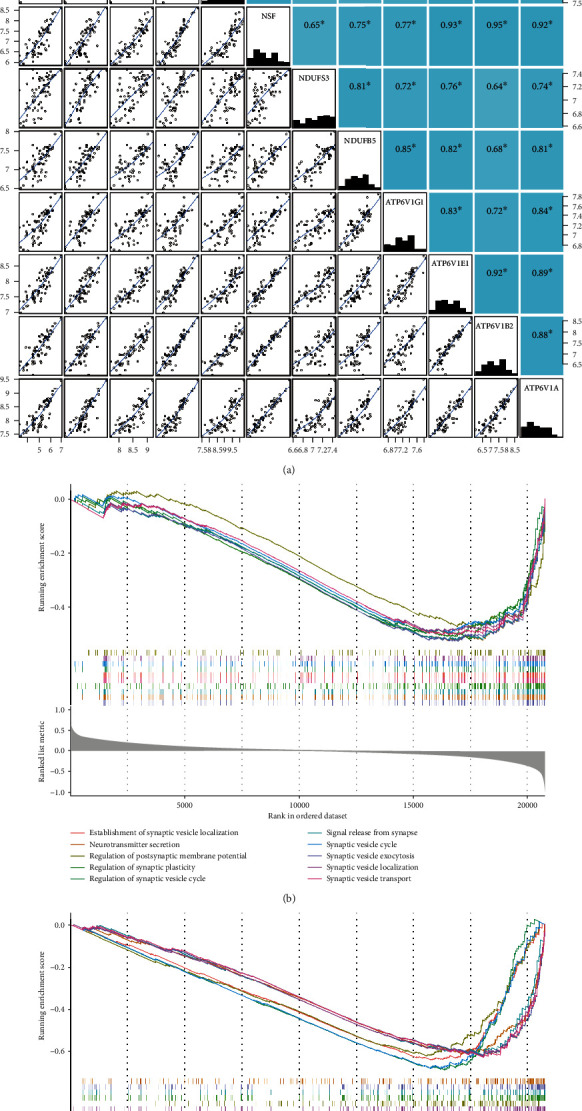
Correlation among genes and gene set enrichment analysis. Correlation of ATP6V1A with signature genes (a): ^∗^*P* < 0.05; blue indicates positive correlation. BP terms of gene set enrichment analysis enriched in AD (b) and ATP6V1A-low (c) groups. AD: Alzheimer's disease; BP: biological processes.

Five signature genes in each cross-talking pathway were determined by PCC analysis (Supplementary Table 1). As shown in Figure 5(a), each signature gene was significantly positively correlated with ATP6V1A expression (*P* < 0.05). In the AD group, the major enrichment of BP involved memory, neurotransmitter secretion, regulation of synaptic plasticity, signal release from synapse, and synaptic vesicle cycle (Figure 5(b)). In the ATP6V1A-low group, the primary enrichment of BP consisted of cellular respiration, neurotransmitter secretion, oxidative phosphorylation, signal release from synapse, and synaptic vesicle cycle (Figure 5(c)).

### 3.5. Cell-Type Specificity of Signature Genes

As shown in Supplementary Figure 1D, a decrease in neurons combined with an increase of OPCs, oligodendrocytes, astrocytes, and endothelial cells were observed, indicating the significant and unique changes in the cell-type composition in AD. As shown in Supplementary Figure 1E, the cell-type specificity of signature genes (including ATP6V1A) was examined by using RNA-seq data derived from different types of cultured brain cells, such as neurons, astrocytes, microglia, endothelial cells, and oligodendrocytes, which presented the downregulation of signature genes in neurons (ATP6V1A, SYT1, SNAP25, NDUFB5, NDUFS3, ATP6V1E1, NSF, and ATP6V1G1), oligodendrocytes (ATP6V1B2), and astrocytes (TUBB2A, TUBA4A, and TUBB3).

## 4. Discussion

In this study, the comparison of ATP6V1A expression was assessed between AD and nondementia controls. Intriguingly, we found downregulation of ATP6V1A in the temporal cortex of AD, a preferential region susceptible to AD neurodegeneration [32]. Further analyses of GSEA involving 20,759 background genes demonstrated that DEGs in AD and ATP6V1A-low cohorts were enriched in neurotransmitter secretion, signal release from synapse, and synaptic vesicle cycle. Of particular note was that these biological processes were possibly related to AD as well as low ATP6V1A expression. In secretory vesicles, V-ATPase was observed to drive neurotransmitter uptake by establishing proton and membrane potential gradients, hence linking the V-ATPase to neurosecretion of synaptic vesicles [33]. Herein, we constructed global regulatory network and coexpression modules of DEGs interacting with ATP6V1A to illustrate the genome-scale mechanism of ATP6V1A in AD pathophysiology.

The results emerging from WGCNA revealed that the blue, brown, and turquoise modules were significantly correlated with AD and ATP6V1A, which were involved in phagosome, oxidative phosphorylation, synaptic vesicle cycle, focal adhesion, and GABAergic synapse. Among them, synaptic vesicle was found to tightly pack and store quanta of neurotransmitter molecules in nerve terminals [34]. Progressive dementia of AD was largely attributed to synaptic defects that were not functioning optimally even before structural deterioration [35]. Both biochemical and stereological evidence of AD presented a better correlation of cognitive decline with reduced synapse density than either A*β* or NFT aggregates, highlighting the fundamental role of synaptic vesicle in AD pathogenesis [36, 37]. Notably, loading of neurotransmitters into synaptic vesicle required a proton gradient, which was precisely dependent on the multisubunit V-ATPase [38]. By contrast, abolishment of proton gradient has dramatic consequences for neurotransmitter release in synaptic vesicle, which should be taken into account when assessing the effects of molecular perturbation of ATP6V1A. Indeed, the effects of V-ATPase on synaptic vesicle were not only restricted to proton pumping and neurosecretion but also implicated in filling downstream and synaptic vesicle fusion [39, 40]. Genomic studies in mice and flies have shown that knockout of ATPase caused impaired presynaptic transmission, along with alterations in the amount and morphology of synapses [41, 42]. These were important processes of V-ATPase deficiency contributing to cognitive impairment and neuronal degeneration [7], consistent with our findings of the participation of low ATP6V1A-mediated synaptic vesicle cycle in AD pathogenesis.

Apart from synaptic vesicle cycle, functional enrichment analysis revealed that ATP6V1A was involved in phagosome and oxidative phosphorylation in AD. In terms of phagosome pathway, it was a step in the degradation processes for lysosome, an acidic and degradative organelle in cells that received and digested all sorts of macromolecules via endocytosis, phagocytosis, and autophagy [43]. During phagocytosis, the particle remained compartmentalized in phagosomes and eventually underwent lysosomal degradation through the phagosome pathway, an important cause of neuronal loss in AD neuropathology [44, 45]. Previous studies showed that A*β* potently activated the phagocytic capacity, by which neurons were eliminated before they died [45, 46]. The results obtained here were supported by inhibition of phagocytosis using cytochalasin D and cyclo-(RGDfV), both of which prevented neuronal loss and unexpectedly increased survival cells in pathologically affected regions of AD [47]. Much evidence has underscored the viewpoint of bioenergetic failure and mitochondrial malfunction in preclinical and established AD [48, 49]. In addition, oxidative phosphorylation has important roles in mitochondrial ATP production and is modulated by respiratory enzyme complexes I–V [50, 51]. In AD model of triple transgenic mice, deregulations of complexes I and IV were tau- and A*β*-dependent, respectively, which was implicated in reduction of mitochondrial proteins [52]. This provided support for the synergistic role of A*β* and tau in perishing mitochondria, leading to reactive oxidative species (ROS) production, bioenergetic exhaustion, and neuronal apoptosis [53, 54]. There was also evidence that mitochondrial DNA mutation in V-ATPase contributed to various defects of oxidative phosphorylation [55, 56]. Inhibition of V-ATPase by Bafilomycin impaired decoupling of oxidative phosphorylation and thus to insufficient energy of neurons [57, 58]; meanwhile, energy could be supplemented by return of oxidative phosphorylation upon reoxygenation, which in turn activated V-ATPase [59]. Likewise, our findings supported the likelihood that low expression of ATP6V1A participated in oxidative phosphorylation and that enhancement of ATP6V1A could be neuroprotective in AD.

The results of logistics regression analysis revealed a causal relationship of AD with elder age and lower ATP6V1A expression, suggesting that either downregulation of ATP6V1A or increase of age might be a pathogenic factor of AD. Evidence in transgenic APP/PS1 model of mice showed that defects of V-ATPase and axonal transport were early pathogenic events deteriorated with age, leading to accumulation of APP and synaptic A*β* [60, 61]. On basis of DEGs that were strongly interacting with ATP6V1A, global regulatory network was constructed to identify the cross-talking pathways of ATP6V1A, including synaptic vesicle cycle, phagosome, and oxidative phosphorylation. The analysis of PCC showed significantly positive correlation of ATP6V1A with signature genes of each cross-talking pathway, which provided computational statistical evidence for the involvement of low ATP6V1A in AD pathogenesis via synaptic vesicle cycle, phagosome, and oxidative phosphorylation. The AUC analysis showed that low ATP6V1A expression accurately predicted AD onset, also implying ATP6V1A to be a potential biomarker of AD. Cell-type proportion analysis exhibited a decrease in neurons along with an increase of OPCs, oligodendrocytes, astrocytes, and endothelial cells, in line with the significant and unique changes in response to misfolded or polymerized A*β* in AD [62]. As supported by experiments using Western blot and qRT-PCR, downregulation of ATP6V1A was identified in MSBB BM36-PHG samples of AD relative to normal controls [31]. Cell-type specific analysis presented the downregulation of signature genes in neurons (ATP6V1A, SYT1, SNAP25, NDUFB5, NDUFS3, ATP6V1E1, NSF, and ATP6V1G1), oligodendrocytes (ATP6V1B2), and astrocytes (TUBB2A, TUBA4A, and TUBB3), supporting the linkage of low ATP6V1A with phagosome of astrocytes, neuronal synaptic vesicle cycle, and oxidative phosphorylation. Further *in vivo* or *in vitro* experiments are encouraged to verify the low ATP6V1A-mediated pathways underlying AD proposed in the current study.

## 5. Conclusions

Overall, the work undertaken in this study suggests that bioinformatics analysis is a promising approach to investigate the complex pathways of ATP6V1A in AD occurrence. Based on our findings, low expression of ATP6V1A was involved in the pathogenesis of AD, which might be mediated via synaptic vesicle cycle, phagosome, and oxidative phosphorylation.

## Figures and Tables

**Table 1:** Logistics regression analysis to detect AD predictors.

Characteristics	AD (*n* = 45)	Control (*n* = 24)	Logistics regression analysis		
			OR	SE	*P* value
Gender (female/male)	25/20	10/14	1.058	0.618	0.928
Age (low/high)	82.7 ± 9.8	71.5 ± 16.9	0.246	0.628	0.026^∗^
ATP6V1A (low/high)	8.04 ± 0.41	8.42 ± 0.46	5.831	0.586	0.003^∗∗^

^∗^ *P* < 0.05; ^∗∗^*P* < 0.01; AD: Alzheimer's disease; OR: odds ratio; SE: standard error.

## Data Availability

The datasets generated and/or analyzed during the current study are available in the GSE118553 repository, https://www.ncbi.nlm.nih.gov/geo/query/acc.cgi?acc=GSE118553.

## Supplementary Materials

Supplementary 1 Supplementary Table 1: signature genes for each cross-talking pathway.

Supplementary 2 Supplementary Figure 1: expression changes of ATP6V1A and cell-type specificity of signature genes. ATP6V1A expression changes in MSBB BM36-PHG samples using Western blot (A and B) and qRT-PCR (C) analyses ([31]; available form doi:10.1016/j.neuron.2020.11.002). Mean change of cell-type proportion by computing the SPV average for the samples after cell-type deconvolution using *BRETIGEA* (D): blue to red indicates the change from a decrease to an increase. Vector addition of squared expression levels of signature genes across five different cell types in AD (E): yellow indicates downregulated expression. BM36-PHG: Brodmann area 36 parahippocampal gyrus; MSBB: Mount Sinai Brain Bank; NL: normal control; SPV: surrogate proportion value; ast: astrocytes; end: endothelial cells; mic: microglia; neu: neurons; oli: oligodendrocytes.
