# Supplementary material for: Downregulation of ATP6V1A Involved in Alzheimer's Disease via Synaptic Vesicle Cycle, Phagosome, and Oxidative Phosphorylation
Source: Oxid Med Cell Longev. 2021 Apr 19;2021:5555634. doi: 10.1155/2021/5555634 (PMC8087993; doi:10.1155/2021/5555634)
Supplement: Supplementary 1 — Supplementary Table 1: signature genes for each cross-talking pathway. [file 5555634.f1.pdf]

**Pathway**	**Genes**	**Signature genes**
Synaptic vesicle cycle	DNM3, RIMS1, STX1B, SYT1, VAMP2, STXBP1, SNAP25, SLC17A7, NSF, ATP6V1H, ATP6V1C1, ATP6V0D2, ATP6V1D, ATP6V1F, ATP6V1B2, ATP6V0B, ATP6V1G2, ATP6V0E2, ATP6V1E1, ATP6V1G1, ATP6V1A	ATP6V1B2, NSF, SYT1, ATP6V1E1, SNAP25
Phagosome	CYC1, COX6B1, ATPSPD, ATP5MC1, ATP5PB, ATP5F1B, ATP6V1H, ATP6V1C1, ATP6V0D2, ATP6V1D, ATP6V1F, ATP6V1B2, ATP6V0B, ATP6V1G2, ATP6V0E2, ATP6V1E1, ATP6V1G1, ATP6V1A, NDUFS8, NDUFS3, NDUFS2, COX7AL, NDUFB5, NDUFS7	ATP6V1E1, ATP6V1B2, ATP6V1G1, NDUFB5, NDUFS3
Oxidative phosphorylation	DYNC1LI1, FCGR3A, DYNC1I1, NYNC1I2, SCARB1, DYNC1H1, HGS, ATP6V1H, ATP6V1C1, ATP6V0D2, ATP6V1D, ATP6V1F, ATP6V1B2, ATP6V0B, ATP6V1G2, ATP6V0E2, ATP6V1E1, ATP6V1G1, ATP6V1A, TUBB4B, TUBB2A, TUBB4A, TUBA4A, TUBB3, TLR2, TAP2, SCEC61A2	ATP6V1B2, ATP6V1E1, TUBA4A, TUBB2A, TUBB3
