# Supplementary figures and images for: Downregulation of ATP6V1A Involved in Alzheimer's Disease via Synaptic Vesicle Cycle, Phagosome, and Oxidative Phosphorylation
Source: Oxid Med Cell Longev. 2021 Apr 19;2021:5555634. doi: 10.1155/2021/5555634 (PMC8087993; doi:10.1155/2021/5555634)

A

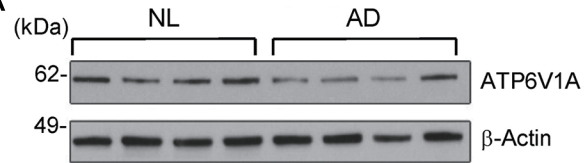

B

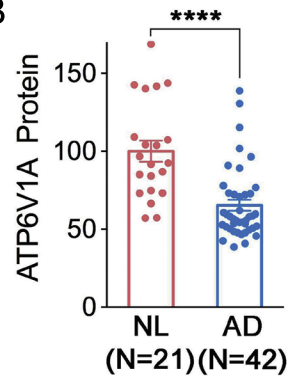

C

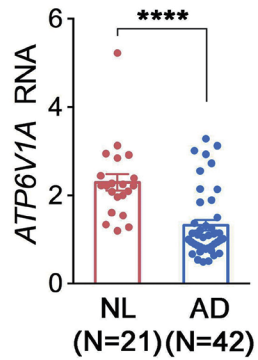

D

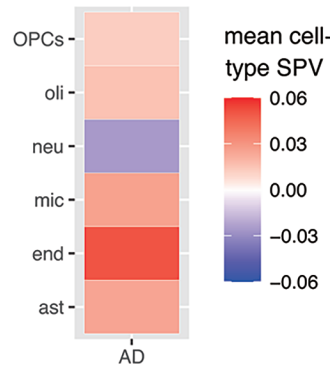

E

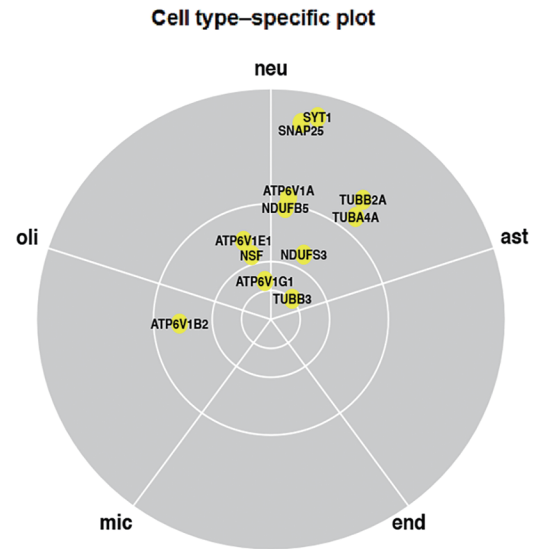

Supplement: Supplementary 2 — Supplementary Figure 1: expression changes of ATP6V1A and cell-type specificity of signature genes. ATP6V1A expression changes in MSBB BM36-PHG samples using Western blot (A and B) and qRT-PCR (C) analyses ([31]; available form doi:10.1016/j.neuron.2020.11.002). Mean change of cell-type proportion by computing the SPV average for the samples after cell-type deconvolution using BRETIGEA (D): blue to red indicates the change from a decrease to an increase. Vector addition of squared expression levels of signature genes across five different cell types in AD (E): yellow indicates downregulated expression. BM36-PHG: Brodmann area 36 parahippocampal gyrus; MSBB: Mount Sinai Brain Bank; NL: normal control; SPV: surrogate proportion value; ast: astrocytes; end: endothelial cells; mic: microglia; neu: neurons; oli: oligodendrocytes. [file 5555634.f2.pdf]
